# Report of Exsection of Shoulder

**Published:** 1890-08

**Authors:** C. H. Cox

**Affiliations:** Savannah, Ga.


### REPORT OF EXSECTION OF SHOULDER.

BY C. H. COX, M. D., SAVANNAH, GA.

May 4th, J., negro boy, fourteen years of age, was brought to my office, six miles from the country, with a gun-shot injury of the shoulder, embracing a space of about two inches or more in diameter, and perforating the deltoid muscle in the back.

The wound was charred, ragged, with pieces of clothing through the same, the boy suffering from loss of blood and the fatigue of the long journey. The shaft of the right humerus was fractured below the surgical neck, with no, or little, injury to the glenoid cavity, the coracoid process, and the axillary artery being intact.

I operated in the afternoon of same day, exsecting the head and sawing off the injured portion of the shaft of the humerus by protruding the same through the anterior wound, removing at least, with the head included, about two inches. After making the wound as free of all septic substances as possible, and irrigating with one to two thousand solution of bichloride, the wound was dried, and sprinkled with iodoform, and dressed in bichloride gauze and cotton in the usual way.

The temperature on the following day was only 100½°, and at no time did his temperature exceed that amount.

He was dressed again on the 8th, with campho-phenique and bichloride gauze. On the 14th he was dressed in like manner, the wound being in a good, red, sweet and healthy condition.

I opened the wound on the 22d and scraped it out thoroughly with a sharp spoon, pared the edges with a pair of scissors, cutting out the hard cicatrices, and I brought the anterior wound together as close as possible, with deep sutures, leaving the posterior wound open for drainage, dressed with campho-phenique and bichloride gauze, no tubing or cat-gut being used for drainage in this or in the first operation, as the posterior wound was sufficiently large. The boy did not have any fever after second operation.

He was dressed again on the 26th, the sutures taken out, with a few drops of pus exuding from one of the holes. With this exception, no pus was in the wound during the entire treatment. The anterior wound almost entirely healed, except where, for want of tissue, the edges of skin could not be brought into good coaptation.

The arm was dressed again on the 30th, the anterior wound well, leaving the now small posterior wound, which healed by the 6th, when the boy was discharged from the hospital well, in one month and two days, having been confined to his bed only five days, and I may as well add, happy in not losing his arm.

In reporting this case to the medical profession, I wish to bring the following points under special attention: In injuries of the shoulder joint, and injuries to other parts, with a good supply of blood, to bring the parts together as soon as all danger from suppuration is passed, thereby obviating a protracted treatment, with a smaller scar, a better result, as the tissues are drawn tighter around the new-made joint, and with more satisfaction to the surgeon and patient.

Iconsider campho-phenique a good substitute for iodoform. We get rid of the disagreeable odor, and at the same time have a drug easy to use, with as good healing properties and greater antiseptic powers, comparatively free from all danger of producing poisoning.

I also think the wound should be dried with absorbent cotton, squeezed before applying campho-phenique. In removing excess of moisture it is not diluted, and at the same time, perhaps, removes other septic properties, and thereby carries out the dry dressing more thoroughly.

